# Knowledge, Attitudes, and Practices Regarding the Long‐Term Health Risks of Energy Drink Consumption Among Adults in Iraq: A Cross‐Sectional Study

**DOI:** 10.1002/hsr2.73484

**Published:** 2026-11-10

**Authors:** Walid Al‐Qerem, Alaa Hussein Alsajri, Anan Jarab, Ahmed Safaa, Eman Sattar, Ruaa Nief, Othman Saad, Omar Nassr, Israa Nather, Judith Eberhardt, Lama Sawaftah, Mayar Sweidan

**Affiliations:** ^1^ Department of Pharmacy, Faculty of Pharmacy Al‐Zaytoonah University of Jordan Amman Jordan; ^2^ College of Pharmacy Madenat Alelem University Baghdad Iraq; ^3^ Department of Clinical Pharmacy, Faculty of Pharmacy Jordan University of Science and Technology Irbid Jordan; ^4^ Department of Pharmacy Al‐Rasheed University College Baghdad Iraq; ^5^ Department of Psychology, School of Social Sciences, Humanities and Law Teesside University Middlesbrough UK

**Keywords:** attitudes, energy drinks, Iraq, knowledge, practices, public health nutrition

## Abstract

**Background and Aims:**

Energy drink consumption is common among young adults, yet evidence on knowledge, attitudes and practices in Iraq remains limited. This study assessed these domains among adults in Iraq and examined associations of knowledge and risk concern with past‐year use and consumption frequency.

**Methods:**

A cross‐sectional online survey was conducted in Iraq from May to November 2025 using a structured Arabic questionnaire distributed through social media. The sample included 1125 adults. Scale reliability and construct validity were assessed using ordinal alpha coefficients and confirmatory factor analysis. Structural equation models examined associations of knowledge and risk concern with past‐year energy drink use and consumption frequency, adjusting for demographic covariates.

**Results:**

Past‐year consumption was reported by 399/1125 participants (35.5%). Of the 1125 participants, 1002 (89.1%) identified arrhythmias, 977 (86.8%) sleep problems and 794 (70.6%) raised blood pressure as potential risks; 468 (41.6%) reported knowing the ingredients. Support for warning labels, awareness campaigns and age restrictions was 1029/1125 (91.5%), 1023/1125 (90.9%) and 974/1125 (86.6%), respectively. Higher knowledge was associated with greater risk concern, which was associated with a lower likelihood of past‐year use and lower consumption frequency among consumers.

**Conclusion:**

Energy drink consumption was common in this sample, alongside strong support for regulation and limited ingredient literacy. The findings support clearer product information and targeted public health education.

## 1 Introduction

Energy drinks are beverages originally developed as performance enhancers and are widely consumed to boost alertness, mental focus, and physical stamina [1]. They typically contain stimulant and “energy‐boosting” ingredients, including caffeine, taurine, herbal extracts, and B vitamins [2]. Energy drink consumption is common among young adults internationally [3], and Iraqi studies have documented consumption in young adult and university populations [4, 5].

Changes in consumption have been documented over time. In the United States, the proportion of adults aged 20–39 years reporting energy drink consumption in a 24‐h dietary recall increased from 0.5% to 5.5% between 2003 and 2016 [6]. An international meta‐analysis estimated past‐year consumption among young adults aged 18–35 years at 45.6% (95% CI: 31.5–59.7), although estimates varied substantially between studies [3]. In Iraq, a survey of 1298 medical students at the University of Mosul found that 30% reported consuming energy drinks, while only 42% knew their ingredients [5].

A growing body of evidence suggests that excessive energy drink consumption may be associated with a range of health consequences, including cardiovascular complications, sleep disturbances, neuropsychiatric effects, and potential adverse effects on the liver and kidneys [7, 8, 9]. Some individuals may be more susceptible to these harms, particularly younger people, those who are caffeine‐naïve, individuals with certain medical conditions, or those taking specific medications [10]. Consumption patterns, including high intake and co‐consumption with alcohol, warrant consideration, although evidence regarding behavioral risks is mixed [10, 11].

Energy drink consumption has been associated with cardiovascular, sleep‐related and other adverse effects, although the strength of evidence differs across outcomes. A meta‐analysis of 17 randomized trials found that acute consumption increased systolic and diastolic blood pressure in healthy adults [12]. Some experimental studies have also identified changes in cardiac repolarisation following high‐volume consumption. For example, a randomized trial [13] reported prolongation of the heart‐rate‐corrected QT interval after participants consumed approximately 946 mL of energy drinks. However, pooled effects on QT intervals were not statistically significant in the meta‐analysis [12], indicating that findings are not consistent across products and study conditions. These trials assessed acute physiological responses, while evidence concerning chronic cardiovascular outcomes remains limited.

Sleep disturbance is another concern, particularly because energy drinks are often consumed to maintain alertness. A systematic review identified insomnia, restlessness, headaches, and gastrointestinal symptoms among reported adverse events; its meta‐analysis of controlled trials supported increased odds of insomnia and jitteriness following consumption [7]. Among Norwegian college and university students, more frequent consumption was associated with shorter sleep duration, lower sleep efficiency and greater sleep disturbance [14]. People experiencing insufficient sleep may also consume energy drinks to counter daytime fatigue.

Less common but potentially serious outcomes include acute kidney injury and hepatitis reported after excessive intake. These case reports raise concerns about heavy consumption but provide limited information about the incidence of organ injury or risks at typical levels of intake [15]. Co‐consumption with alcohol presents a separate concern: a randomized experiment found that adding an energy drink increased stimulation without reversing alcohol‐related impairment of response inhibition [16]. Risk communication should therefore distinguish the potential consequences of high intake and co‐consumption from the less certain long‐term effects of habitual use.

Previous Iraqi research has documented energy drink consumption [4]. However, evidence integrating knowledge, risk concern, consumption practices and support for preventive measures remains limited. Understanding these factors can inform locally relevant educational content and public health measures.

Interventions intended to reduce energy drink consumption have included education, behavioral support, warning labels and restrictions on marketing and availability. Educational approaches aim to improve understanding of product ingredients and potential harms. In a study involving 443 medical students in India, 4 weekly educational sessions were followed by reductions in self‐reported energy drink consumption. However, the absence of a comparison group and the short follow‐up limited conclusions about intervention effectiveness [17]. Behavioral support may also be relevant when consumption has become habitual. A small randomized trial among adults seeking help for problematic caffeine use found that a manual‐based program incorporating gradual reduction produced decreases in caffeine intake that persisted for 20 weeks after treatment [18]. Most participants primarily consumed coffee, however, so the findings cannot be assumed to apply directly to energy drink consumers.

Warning labels provide another means of communicating potential harms at the point of consumption, although evidence of their behavioral effects remains limited. In a randomized online experiment involving 343 Australian adult energy drink consumers, graphic warnings concerning cardiac effects or weight gain did not significantly affect overall intentions to reduce consumption compared with a no‐warning condition. Cardiac warnings attracted greater attention than weight‐gain warnings, but attention to a message should not be equated with a reduction in intake [19]. This distinction supports evaluating both how consumers understand health messages and whether those messages lead to sustained changes in behavior.

Regulatory approaches address the commercial environment in which consumption occurs. In Saudi Arabia, measures announced in 2014 included restrictions on advertising, free promotional distribution and sales in specified educational, health and sports settings, together with warning statements on products [20]. Poland subsequently introduced restrictions on sales to people younger than 18 years and on sales in schools and vending machines from January 2024 [21]. These examples illustrate measures adopted to limit exposure and access, although their implementation alone does not demonstrate reductions in consumption or adverse health outcomes.

The relevance of these approaches to Iraq depends partly on local consumption practices, understanding of health risks and acceptance of preventive measures. Examining knowledge alongside risk concern and consumption can help identify priorities for educational content, while assessing support for warning labels, awareness campaigns and age restrictions can inform discussions about locally acceptable public health responses.

Therefore, this study aimed to assess levels of knowledge, attitudes, and practices related to energy drink consumption among adults in Iraq, with a particular focus on perceived health effects, sources of information, and support for public health regulations.

## 2 Methods

### 2.1 Study Design and Participants

This cross‐sectional study collected data from May to November 2025. A structured, self‐administered questionnaire was developed in Google Forms and distributed by the research team to adults living in Iraq through Facebook, WhatsApp, Instagram, and Telegram. Eligibility was assessed through screening questions on age and current residence; only respondents indicating that they were adults residing in Iraq could proceed to the main questionnaire. Google Forms was configured to require account sign‐in and limit submissions to one response per Google account. Submitted responses were checked for internal consistency and response patterns suggesting potential duplication.

Convenience sampling was selected because it provided a feasible means of recruiting adults residing in Iraq within the available time and resources. The questionnaire was distributed through several social‐media platforms, including groups serving different geographical areas, to broaden recruitment opportunities beyond a single locality. The practicality of online recruitment was supported by widespread internet use in Iraq, where approximately 81.5% of the population used the internet in 2024, according to International Telecommunication Union data reported by the World Bank [22].

Although all adults residing in Iraq were eligible, recruitment placed particular emphasis on younger adults. This focus reflected evidence of higher energy drink consumption among younger than middle‐aged adults in nationally representative U.S. data [6]. Online recruitment was also appropriate for reaching younger people given their high internet use in the region: 86% of individuals aged 15–24 years in the Arab States used the internet in 2024, compared with 67% of the remaining population [23].

The self‐administered format allowed participants to complete the questionnaire without an interviewer present, which may reduce pressures to provide socially desirable responses [24]. Confidential participation was supported by the absence of direct personal identifiers and restricted access to survey responses. Nevertheless, widespread internet use and dissemination across geographical areas did not eliminate self‐selection or coverage bias [25].

The opening page of the survey explained the study purpose, the voluntary nature of participation, confidentiality arrangements, and participants’ right to discontinue the survey before submitting their responses. Electronic informed consent was obtained through a mandatory agreement checkbox before participants could proceed to the questionnaire. No payment or other incentives were offered. Ethical approval was granted by the Institutional Review Board and the Deanship of Research at Al‐Rasheed University College (reference number: RUCPD10012603). The study was conducted in accordance with the Declaration of Helsinki.

Survey responses were downloaded from Google Forms and stored on a password‐protected computer. The online responses were subsequently deleted, while the downloaded dataset was retained. Access to the password‐protected survey account and downloaded dataset was restricted to the primary investigators. The questionnaire did not collect names, email addresses, or other direct personal identifiers.

The questionnaire took approximately 10 min to complete. All applicable questions were mandatory, preventing submission of questionnaires with unanswered applicable items. Submitted responses underwent internal consistency and duplicate‐pattern checks, and no submissions were excluded on these grounds.

### 2.2 Tool Development and Validation

Following an extensive review of previously published studies on the health risks associated with energy drink consumption, the study questionnaire was developed [26, 27, 28]. The questionnaire included four sections. The first section collected participants’ sociodemographic characteristics, including age, gender, education level, and occupation. The second section assessed participants’ knowledge of energy drinks. The third section evaluated attitudes, risk awareness, and perceived vulnerability related to energy drinks. The final section examined participants’ energy drink consumption practices.

Content validity was assessed by an expert panel consisting of an endocrinologist, an internist, two pharmacists, and a clinical pharmacist. The questionnaire was translated using a forward–backward translation procedure. Two independent translators first produced two Arabic versions. The research team then reviewed both versions and resolved discrepancies to produce a single agreed‐upon Arabic version. This version was back‐translated into English and compared with the original questionnaire, and all versions were reviewed to identify and address any remaining discrepancies. The translation process followed Brislin's principles for cross‐cultural research [29].

A pilot study with 30 Iraqi adults was conducted to assess questionnaire clarity and internal consistency. Informed consent was obtained before participation. Participants were asked to provide feedback on item clarity, wording, and perceived difficulty. Pilot data were not included in the final analysis.

The study was guided by the knowledge–attitudes–practices (KAP) framework and the Health Belief Model (HBM) [30, 31]. The KAP framework guided the organization of the questionnaire into knowledge of energy drink health risks, attitudes and beliefs, and reported consumption practices. The HBM informed the assessment of perceived susceptibility, health‐threat appraisal, confidence in avoiding harm, and potential cues to action [32].

Perceived susceptibility was addressed through beliefs about personal harm from regular consumption and the vulnerability of children and adolescents. Health‐threat appraisal encompassed concern about long‐term consequences and beliefs about harms associated with high intake and co‐consumption with alcohol. Self‐efficacy‐related beliefs were addressed through confidence in avoiding health risks by stopping consumption. Questions about warning‐label exposure, the influence of warnings on consumption decisions, and health‐information sources addressed potential cues to action. Additional items examined perceived performance benefits, consumption motives, information needs, and support for public‐health measures.

In the structural models, the attitude/regulation, risk‐perception, and vulnerability scores served as indicators of a broader latent risk concern construct. Knowledge was examined in relation to consumption both directly and indirectly through risk concern, with adjustment for participant characteristics. Past‐year consumption and consumption frequency among consumers were evaluated in separate models.

### 2.3 Sample Size Calculation

The minimum sample size was estimated using the single‐population proportion formula, *n* = Z^2^P(1 − P)/d^2^, where *Z* = 1.96 corresponded to a 95% confidence level and *d* = 0.04 represented an absolute precision of four percentage points. An anticipated proportion of 0.50 was selected because no suitable prior estimate was available; this conservative assumption maximized the required sample size. Substitution yielded *n* = (1.96^2^ × 0.50 × 0.50)/0.04^2^ = 600.25, which was rounded upward to 601 participants.

No finite‐population correction or numerical adjustment for design effect or anticipated non‐response was applied to this minimum. Recruitment deliberately exceeded the minimum to allow for potential design effects, resulting in a final sample of 1125 participants. Given the convenience sampling approach, the calculation served as a recruitment planning benchmark.

### 2.4 Statistical Analysis

All analyses were conducted in R, with version 4.5.2 recorded in the saved analysis workspace. Confirmatory factor analysis and structural equation modeling were performed using lavaan version 0.6‐19 [33], and reliability analyses used psych. Statistical significance was assessed using two‐sided tests with an alpha level of 0.05; significance was determined from unrounded P values. Descriptive statistics were used to summarize participant characteristics and questionnaire responses. Internal consistency for each scale was evaluated using ordinal reliability coefficients (alpha) based on polychoric correlations; this approach was chosen because the study data were ordinal [34].

Before testing the structural models, confirmatory factor analyses (CFAs) were conducted to evaluate the measurement properties of each scale. Model adequacy was assessed using standardized factor loadings and global fit indices. To reduce model complexity and align the analysis with the study's focus on construct‐level relationships, scale scores were computed before structural modeling. For each multi‐item domain, item responses were combined into a composite score by calculating the mean of the items. For the knowledge domain, binary‐scored items (correct/incorrect) were summed to generate a total knowledge score. For the attitude/regulation, risk perception, and vulnerability domains, items were coded so that higher scores reflected stronger support for regulatory measures, greater perceived risk, and greater perceived vulnerability, respectively. The knowledge total and the composite attitude/regulation, risk perception, and vulnerability scores were standardized before inclusion in the structural models.

A latent “risk concern” construct was then specified, indicated by the composite scores for attitude/regulation, risk perception, and vulnerability. The measurement component for this latent construct was evaluated within the SEM by examining standardized factor loadings and the explained variance for each indicator.

SEM was used to examine the hypothesized relationships among the study constructs while adjusting for age group, sex, residence, education, medical‐field status, smoking status, chronic disease, and employment status. This approach was used to capture shared variance as a single underlying construct while retaining measurement error at the indicator level. Both SEMs were planned before analysis: one included the full sample, and the other included the past‐year‐consumer subsample. In the full sample (*N* = 1125), the primary outcome was past 12‐month energy drink use (binary), modeled as an ordered categorical outcome with a probit link using the weighted least squares mean‐ and variance‐adjusted estimator (WLSMV/DWLS). In the past‐year‐consumer subsample (*n* = 399), consumption frequency was analyzed as an ordinal outcome using the same WLSMV/DWLS estimator with a probit link.

Missing data were handled using pairwise present information to estimate polychoric and polyserial associations under WLSMV/DWLS, and the effective sample size contributing to each model was reported. Model adequacy was evaluated using the comparative fit index (CFI), Tucker‐Lewis index (TLI), root mean square error of approximation (RMSEA), and standardized root mean square residual (SRMR). Scaled CFI, TLI, and RMSEA were reported for the structural models. For interpretation of the structural paths, explained variance (R^2^) was reported for risk concern and each outcome. For ordered outcomes, R^2^ was interpreted as the proportion of variance explained in the underlying continuous latent response variable, rather than in the observed ordinal categories. Structural associations were reported using unstandardized coefficients (B), robust standard errors (SE), and standardized coefficients (β), with two‐sided Wald z tests. For the full‐sample model, 95% confidence intervals for B were also reported.

## 3 Results

A total of 1125 participants met the inclusion criteria and provided complete responses to the applicable questionnaire items. Demographic characteristics are presented in Table 1. The most represented age group was 18–25 years (*n* = 895, 79.6%), followed by 26–30 years (*n* = 129, 11.5%) and ≥ 31 years (*n* = 101, 9.0%). Most participants were female (*n* = 737, 65.5%). Regarding education, the majority held a bachelor's degree (*n* = 868, 77.2%), while 257 participants (22.8%) had a high school education or less. Most participants resided in urban areas (*n* = 821, 73.0%). The majority were non‐smokers (*n* = 958, 85.2%), and 997 (88.6%) reported no chronic disease.

**Table 1:** Demographic characteristics of study's participants (*N* = 1125).

		Count (%)
Age	18–25	895 (79.6%)
	26–30	129 (11.5%)
	≥ 31	101 (9%)
Gender	Female	737 (65.5%)
	Male	388 (34.5%)
Place of residence	Rural	304 (27%)
	Urban	821 (73%)
Education level	High school or less	257 (22.8%)
	University degree	868 (77.2%)
	Postgraduate degree	0 (0%)
Working or studying in the medical field	No	310 (27.6%)
	Yes	815 (72.4%)
Smoking status	Non‐smoker	958 (85.2%)
	Smoker	167 (14.8%)
Chronic disease	No	997 (88.6%)
	Yes	128 (11.4%)
Occupation	Unemployed or homemaker	72 (6.4%)
	Freelancer	81 (7.2%)
	Student	783 (69.6%)
	Employed	189 (16.8%)

Table 2 presents knowledge about energy drinks among the 1125 participants. Most respondents correctly identified that energy drinks may raise blood pressure (*n* = 794, 70.6%), cause an irregular heartbeat (*n* = 1002, 89.1%), increase the risk of heart disease when consumed daily (*n* = 997, 88.6%), and lead to sleep problems with long‐term use (*n* = 977, 86.8%). Similarly, 946 participants (84.1%) recognized that energy drinks may harm the kidneys and liver, and that mixing them with alcohol increases health risks. However, awareness of energy drink contents was low: only 468 participants (41.6%) reported knowing their ingredients.

**Table 2:** Participants’ knowledge and perceptions of energy drinks (*N* = 1125).

	Incorrect	Correct
Energy drinks can raise blood pressure	331 (29.4%)	794 (70.6%)
Energy drinks may cause an irregular heartbeat (arrhythmia)	123 (10.9%)	1002 (89.1%)
Consuming energy drinks daily increases the risk of heart disease	128 (11.4%)	997 (88.6%)
Energy drinks can cause anxiety and nervousness	333 (29.6%)	792 (70.4%)
Long‐term consumption of energy drinks may lead to sleep problems such as insomnia	148 (13.2%)	977 (86.8%)
Energy drinks may harm the kidneys and liver over time	179 (15.9%)	946 (84.1%)
Mixing energy drinks with alcohol increases health risks	179 (15.9%)	946 (84.1%)
Do you know the content of Energy drinks	657 (58.4%)	468 (41.6%)

Table 3 summarizes attitudes, risk awareness, and perceived vulnerability regarding energy drinks among the 1125 participants. For attitudes toward regulation, most participants agreed that warning labels about heart, liver, and kidney risks should be mandatory (*n* = 1029, 91.5%), followed by agreement that public awareness campaigns are necessary (*n* = 1023, 90.9%) and that energy drinks should have age restrictions on purchase (*n* = 974, 86.6%).

**Table 3:** Attitudes, risk awareness, and perceived vulnerability regarding energy drinks (*N* = 1125).

	Agree	Neutral	Disagree
**Attitudes**				
I am concerned about the long‐term health risks of energy drinks.		936 (83.2%)	141 (12.5%)	48 (4.3%)
Energy drinks should have age restrictions on purchase.		974 (86.6%)	98 (8.7%)	53 (4.7%)
Warning labels about heart, liver, and kidney risks should be mandatory.		1029 (91.5%)	59 (5.2%)	37 (3.3%)
Public awareness campaigns about energy drink risks are necessary.		1023 (90.9%)	79 (7%)	23 (2%)
**Risk awareness**				
I believe consuming energy drinks regularly could harm my health.		924 (82.1%)	160 (14.2%)	41 (3.6%)
I am aware that energy drinks may cause sleep problems or insomnia.		981 (87.2%)	115 (10.2%)	29 (2.6%)
Energy drinks can be dangerous if consumed in large quantities.		1052 (93.5%)	52 (4.6%)	21 (1.9%)
Mixing energy drinks with alcohol is more harmful than drinking them alone.		1016 (90.3%)	73 (6.5%)	36 (3.2%)
Energy drinks are addictive and may lead to dependency.		847 (75.3%)	218 (19.4%)	60 (5.3%)
I believe energy drinks are as risky as smoking or excessive caffeine consumption.		811 (72.1%)	240 (21.3%)	74 (6.6%)
Perceived Vulnerability and Responsibility				
I am worried about the potential long‐term health effects of energy drinks on young people (e.g., teenagers, university students).		994 (88.4%)	81 (7.2%)	50 (4.4%)
Energy drink consumption poses a significant public health risk in Iraq.		973 (86.5%)	87 (7.7%)	65 (5.8%)
Children and adolescents are especially vulnerable to the harmful effects of energy drinks.		1041 (92.5%)	41 (3.6%)	43 (3.8%)
Energy drink advertisements encourage unhealthy consumption habits among young people.		993 (88.3%)	75 (6.7%)	57 (5.1%)
I believe energy drink consumption should be restricted or regulated in Iraq (e.g., age limits, warning labels).		1023 (90.9%)	56 (5%)	46 (4.1%)
Stronger public awareness campaigns are needed to educate people about the risks of energy drinks.		1024 (91%)	70 (6.2%)	31 (2.8%)
I feel confident in my ability to avoid the health risks associated with energy drinks if I choose to stop consuming them.		962 (85.5%)	125 (11.1%)	38 (3.4%)

Regarding risk awareness, most participants agreed that energy drinks can be dangerous when consumed in large quantities (*n* = 1052, 93.5%), that mixing them with alcohol is more harmful (*n* = 1016, 90.3%), and that they may cause sleep problems or insomnia (*n* = 981, 87.2%). In addition, a substantial proportion perceived energy drinks as addictive (*n* = 847, 75.3%).

For perceived vulnerability and responsibility, most participants agreed that children and adolescents are especially vulnerable to harmful effects (92.5%), that stronger public awareness campaigns are needed to educate people about energy drink risks (91.0%), and that energy drinks should be restricted or regulated (90.9%).

As shown in Table 4, 726 of the 1125 participants (64.5%) reported no energy drink consumption in the past 12 months. Among the 399 participants who reported use, the most common intake frequency was less than once per month (*n* = 177, 44.4%), and more than half reported consuming less than 250 mL per session (*n* = 235, 58.9%). A total of 156 consumers (39.1%) reported having consumed more than two energy drinks in a day, and 26 (6.5%) reported mixing energy drinks with alcohol. Regarding label awareness, 91 (22.8%) past‐year consumers reported rarely reading ingredient labels.

**Table 4:** Practices related to energy drink consumption (*N* = 1125).

		Count (%)
Have you consumed an energy drink in the past 12 months?	No	726 (64.5%)
	Yes	399 (35.5%)
How often do you consume energy drinks? *	Less than once a month	177 (44.4%)
	1–3 times/month	112 (28.1%)
	1–3 times/week	44 (11%)
	4 or more times/week	66 (16.5%)
How much do you usually drink per session? *	< 250 mL	235 (58.9%)
	250–500 mL	151 (37.8%)
	> 500 mL	13 (3.3%)
Do you mix energy drinks with alcohol? *	No	373 (93.5%)
	Yes	26 (6.5%)
Have you ever exceeded the recommended maximum of two energy drinks per day? *	No	243 (60.9%)
	Yes	156 (39.1%)
Do you read the content label (ingredients, caffeine amount) before consuming energy drinks? *	Always	107 (26.8%)
	Sometimes	151 (37.8%)
	Rarely	91 (22.8%)
	Never	50 (12.5%)
Do you notice and read the warning labels on energy drink cans (e.g., “Not for children or people with heart conditions”)? *	Always	138 (34.6%)
	Sometimes	147 (36.8%)
	Rarely	65 (16.3%)
	Never	49 (12.3%)
Do warning labels influence your decision to consume energy drinks? *	Yes	184 (46.1%)
	Not sure	97 (24.3%)
	No	118 (29.6%)

*Note:* (*) These items were answered only by participants who reported consuming energy drinks in the past 12 months (*n* = 399). Percentages were calculated within this subgroup.

Figure 1 illustrates participants’ experiences after consuming energy drinks. The most commonly reported symptoms were heart palpitations or an irregular heartbeat (196, 49.12%), followed by trouble sleeping (170, 42.61%). Other reported symptoms included anxiety or restlessness (84, 21.05%), nausea or stomach discomfort (79, 19.80%), and headaches (78, 19.55%). The fewest participants reported signs of liver or kidney stress (39, 9.77%). Eighty‐two participants (20.55%) reported none of the listed symptoms.

**Figure 1:**
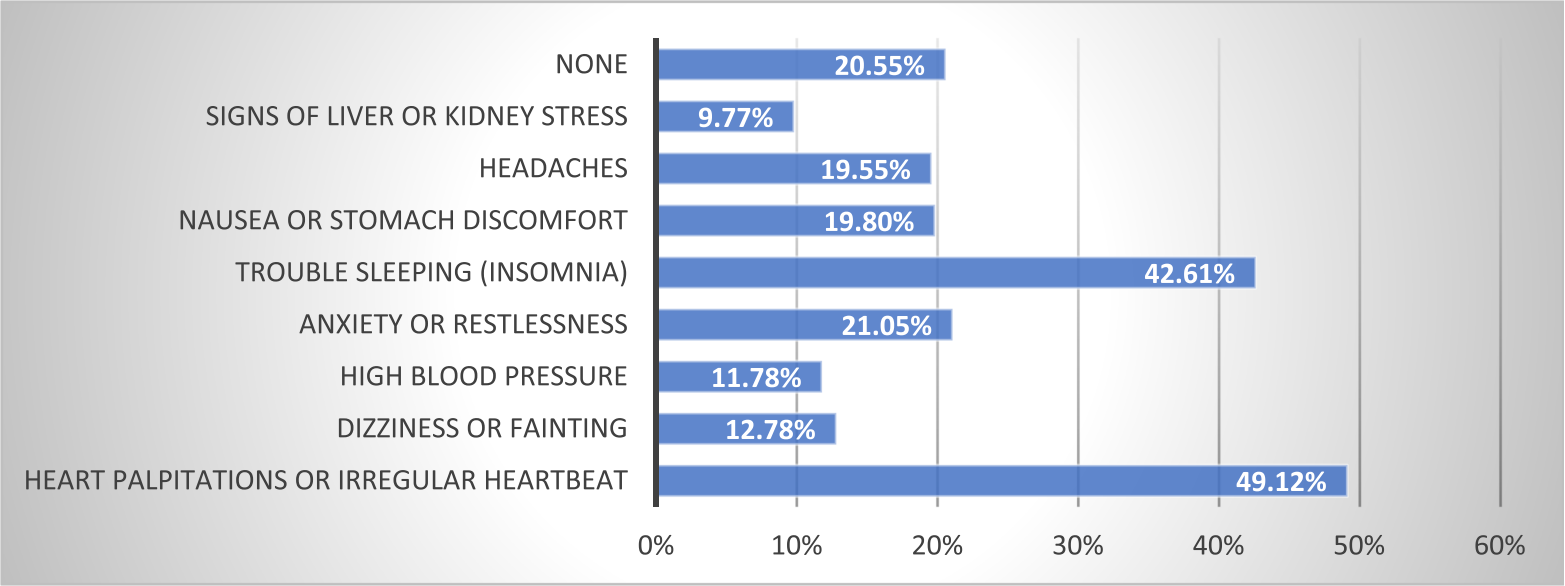
Self‐reported symptoms and perceived health problems after energy drink consumption among past‐year consumers (*n* = 399). Percentages were calculated using these 399 respondents; participants could select more than one response.

### 3.1 Scale Assessment and Construct Validity

Internal consistency was acceptable across the study scales, with ordinal alpha values ranging from 0.83 for the vulnerability scale to 0.91 for the objective knowledge scale. CFA supported the intended measurement structure: standardized factor loadings were statistically significant, and no indicators fell below the prespecified threshold for problematic loadings (< 0.40) (Table 5). Loadings ranged from 0.66 to 0.93 for the knowledge indicators, 0.76 to 0.94 for the attitudinal/regulatory indicators, 0.61 to 0.88 for the perceived risk indicators, and 0.69 to 0.90 for the vulnerability indicators.

**Table 5:** Confirmatory factor analysis results for study scales (standardized factor loadings).

Scale	Item	Est	SE	*p*	Std_loading
Objective Knowledge Scale	Energy drinks can raise blood pressure	0.703	0.031	<0.001	0.703
	Energy drinks may cause an irregular heartbeat (arrhythmia)	0.931	0.023	<0.001	0.931
	Consuming energy drinks daily increases the risk of heart disease	0.863	0.027	<0.001	0.863
	Energy drinks can cause anxiety and nervousness	0.708	0.029	<0.001	0.708
	Long‐term consumption of energy drinks may lead to sleep problems such as insomnia	0.769	0.033	<0.001	0.769
	Energy drinks may harm the kidneys and liver over time	0.741	0.033	<0.001	0.741
	Mixing energy drinks with alcohol increases health risks	0.658	0.039	<0.001	0.658
Attitudes/regulation scale	Concerned about long‐term health risks (reverse‐coded to higher = more concern)	0.777	0.030	<0.001	0.777
	Age restrictions should apply (reverse‐coded to higher = more restrictive)	0.755	0.033	<0.001	0.755
	Mandatory warning labels (reverse‐coded to higher = more supportive)	0.910	0.025	<0.001	0.910
	Need for awareness campaigns (reverse‐coded to higher = more supportive)	0.936	0.021	<0.001	0.936
Perceived risk scale	Regular consumption could harm my health	0.613	0.040	<0.001	0.613
	Awareness that energy drinks may cause sleep problems/insomnia	0.767	0.035	<0.001	0.767
	Energy drinks can be dangerous if consumed in large quantities	0.878	0.038	<0.001	0.878
	Mixing energy drinks with alcohol is more harmful than drinking them alone	0.747	0.040	<0.001	0.747
	Energy drinks are addictive and may lead to dependency	0.639	0.034	<0.001	0.639
	Energy drinks are as risky as smoking/excessive caffeine	0.652	0.034	<0.001	0.652
Vulnerability scale	Worried about long‐term effects on young people	0.823	0.025	<0.001	0.823
	Energy drinks are a significant public health risk in Iraq	0.832	0.023	<0.001	0.832
	Children/adolescents are especially vulnerable to the effects of energy drinks	0.863	0.025	<0.001	0.863
	Advertisements encourage unhealthy habits	0.854	0.022	<0.001	0.854
	Consumption should be regulated (age limits, warning labels)	0.904	0.019	<0.001	0.904
	Stronger public awareness campaigns are needed	0.899	0.020	<0.001	0.899
	Confident I can avoid risks if I stop consuming energy drinks	0.692	0.035	<0.001	0.692

### 3.2 Latent Risk/Concern Factor

In the full sample, the latent risk concern construct was well captured by the three scale scores, with strong standardized loadings for attitudes/regulation (β = 0.730), risk perception (β = 0.746), and vulnerability (β = 0.824). Among participants who reported energy drink consumption in the past 12 months, the latent risk concern factor again showed strong standardized loadings for attitudes/regulation (β = 0.773), risk perception (β = 0.690), and vulnerability (β = 0.804).

### 3.3 SEM for Past‐Year Consumption as the Outcome

The SEM for past‐year consumption showed acceptable fit to the data (CFI = 0.937, TLI = 0.985, RMSEA = 0.055, SRMR = 0.019; Table 6). Higher knowledge scores were associated with greater risk concern (β = 0.535, *p* < 0.001; Table 6). Participants aged ≥ 31 years reported lower risk concern than those aged 18–25 (β = −0.114, *p* < 0.001), while higher education was associated with greater risk concern (β = 0.116, *p* < 0.001). Smoking status was inversely associated with risk concern (β = −0.099, *p* < 0.001), and chronic disease showed a small negative association (β = −0.054, *p* = 0.05). Employment (vs. unemployed or homemaker) was positively associated with risk concern (β = 0.128, *p* = 0.005); other demographic covariates were not statistically significant.

**Table 6:** Structural equation model of factors associated with past 12‐month energy drink use.

Variable	*B*	SE	95% CI for B	*p*	β
Risk concern (latent)					
**Knowledge score**	**0.689**	**0.031**	**0.628 to 0.750**	**<0.001**	**0.535**
Age 26–30 (vs. 18–25)	−0.153	0.133	−0.413 to 0.108	0.25	−0.038
**Age** ≥ **31 (vs. 18–25)**	**−0.515**	**0.137**	**−0.784 to −0.247**	**<0.001**	**−0.114**
Male (vs. female)	−0.069	0.086	−0.237 to 0.099	0.42	−0.025
Urban (vs. rural)	−0.026	0.080	−0.183 to 0.131	0.75	−0.009
**Higher education (vs. lower)**	**0.355**	**0.084**	**0.191 to 0.518**	**<0.001**	**0.116**
Medical field (yes vs. no)	−0.064	0.081	−0.222 to 0.093	0.42	−0.022
**Smoker (vs. non‐smoker)**	**−0.358**	**0.100**	**−0.554 to −0.161**	**<0.001**	**−0.099**
**Chronic disease (Yes vs. No)**	**−0.218**	**0.109**	**−0.432 to −0.003**	**0.05**	**−0.054**
Freelancer (vs. unemployed/homemaker)	−0.279	0.167	−0.607 to 0.048	0.09	−0.056
Student (vs. unemployed/homemaker)	0.128	0.145	−0.156 to 0.413	0.38	0.046
**Employed (vs. unemployed/homemaker)**	**0.440**	**0.155**	**0.136 to 0.743**	**0.005**	**0.128**
Past 12‐month energy drink use					
**Risk concern (latent)**	**−0.259**	**0.038**	**−0.333 to −0.185**	**<0.001**	**−0.292**
**Knowledge score**	**0.127**	**0.052**	**0.025 to 0.229**	**0.01**	**0.112**
Age 26–30 (vs. 18–25)	−0.141	0.161	−0.456 to 0.175	0.38	−0.039
**Age** ≥ **31 (vs. 18–25)**	**−0.460**	**0.189**	**−0.831 to −0.089**	**0.02**	**−0.115**
**Male (vs. female)**	**0.525**	**0.096**	**0.337 to 0.713**	**<0.001**	**0.219**
**Urban (vs. rural)**	**0.256**	**0.096**	**0.068 to 0.443**	**0.007**	**0.100**
Higher education (vs. lower)	−0.062	0.108	−0.273 to 0.148	0.56	−0.023
Medical field (yes vs. no)	−0.086	0.105	−0.292 to 0.119	0.41	−0.034
**Smoker (vs. non‐smoker)**	**0.881**	**0.128**	**0.629 to 1.133**	**<0.001**	**0.275**
Chronic disease (Yes vs. No)	0.138	0.125	−0.107 to 0.384	0.27	0.039
**Freelancer (vs. unemployed/homemaker)**	**0.459**	**0.232**	**0.005 to 0.913**	**0.05**	**0.104**
**Student (vs. unemployed/homemaker)**	**0.524**	**0.199**	**0.135 to 0.914**	**0.008**	**0.212**
Employed (vs. unemployed/homemaker)	0.294	0.234	−0.165 to 0.752	0.21	0.096
**Indirect and total associations of knowledge with consumption**					
**Knowledge → Risk concern → Consumption**	**−0.178**	**0.027**	**−0.230 to −0.126**	**<0.001**	**−0.156**
Total association of knowledge with consumption	−0.051	0.045	−0.139 to 0.037	0.25	−0.045

*Note:* Bold values indicates significance at p value less than 0.05.

Risk concern was inversely associated with reporting past 12‐month energy drink use (β = −0.292, *p* < 0.001), whereas knowledge showed a small direct positive association with consumption (β = 0.112, *p* = 0.01). Male participants had a higher likelihood of past‐year use than female participants (β = 0.219, *p* < 0.001), and urban residence was positively associated with consumption (β = 0.100, *p* = 0.007). Smoking was also strongly associated with past‐year use (β = 0.275, *p* < 0.001). Compared with the unemployed or homemaker group, freelancing (β = 0.104, *p* = 0.05) and student status (β = 0.212, *p* = 0.008) were associated with higher consumption, while older age (≥ 31 vs. 18–25) was inversely associated with consumption (β = −0.115, *p* = 0.02).

Consistent with the opposing direct and indirect associations, the indirect association of knowledge with consumption through risk concern was negative and statistically significant (β = −0.156, *p* < 0.001), whereas the total association of knowledge was not significant (β = −0.045, *p* = 0.25). The model explained 39.8% of the variance in risk concern and 28.2% of the variance in the underlying latent response for past‐year consumption.

B, unstandardized coefficient; SE, robust standard error; CI, confidence interval; beta, standardized coefficient. Coefficients for past‐year consumption are on the probit latent‐response scale. Bold indicates *p* < 0.05 based on the unrounded *p*‐value.

Among participants reporting past‐year consumption, the SEM for consumption frequency showed good fit (CFI = 0.936, TLI = 0.985, RMSEA = 0.041, SRMR = 0.021). Knowledge was positively associated with risk concern (β = 0.577, *p* < 0.001). Participants aged ≥ 31 years reported lower risk concern than those aged 18–25 (β = −0.174, *p* = 0.001), whereas higher education was associated with greater risk concern (β = 0.142, *p* = 0.001). No other covariates were statistically significant; estimates for freelancing (vs unemployed or homemaker; β = −0.190, *p* = 0.05) and working or studying in a medical field (β = −0.086, *p* = 0.07) were negative.

In contrast, risk concern was inversely associated with consumption frequency (β = −0.242, *p* < 0.001). None of the demographic covariates were significantly associated with consumption frequency; the estimate for urban residence was positive (β = 0.106, *p* = 0.07). The indirect association of knowledge with consumption frequency through risk concern was negative and statistically significant (β = −0.140, *p* < 0.001). The total association of knowledge with consumption frequency was also negative and statistically significant (β = −0.137, *p* = 0.02). The model explained 44.6% of the variance in risk concern and 10.0% of the variance in the underlying latent response for consumption frequency (Table 7).

**Table 7:** SEM predictors of risk concern and consumption frequency among past‐year energy drink consumers.

Predictors	*B*	SE	*p*	β
Risk concern (latent)				
Knowledge score	0.787	0.060	<0.001	0.577
Age 26–30 versus 18–25	−0.229	0.226	0.311	−0.054
Age ≥ 31 versus 18–25	−0.827	0.257	0.001	−0.174
Male versus Female	0.065	0.155	0.675	0.024
Urban versus Rural	−0.155	0.153	0.309	−0.050
University/Postgraduate degree versus ≤High school	0.427	0.134	0.001	0.142
Working in the medical field: Yes versus No	−0.247	0.136	0.070	−0.086
Smoker: Yes versus No	0.022	0.167	0.895	0.008
Chronic disease: Yes versus No	−0.223	0.180	0.216	−0.057
Freelancer versus Unemployed/homemaker	−0.848	0.437	0.052	−0.190
Student versus Unemployed/homemaker	−0.336	0.391	0.390	−0.115
Employed versus Unemployed/homemaker	−0.151	0.430	0.725	−0.041
Consumption frequency				
**Risk concern (latent)**	**−0.187**	**0.050**	**<0.001**	**−0.242**
Knowledge score	0.003	0.072	0.972	0.002
Age 26–30 versus 18–25	0.033	0.217	0.880	0.010
Age ≥ 31 versus 18–25	−0.203	0.282	0.471	−0.056
Male versus Female	0.169	0.133	0.205	0.082
Urban versus Rural	0.256	0.142	0.073	0.106
University/Postgraduate degree versus ≤High school	−0.063	0.146	0.665	−0.027
Working in the medical field: Yes versus No	−0.166	0.143	0.245	−0.075
Smoker: Yes versus No	0.223	0.145	0.124	0.099
Chronic disease: Yes versus No	0.007	0.175	0.969	0.002
Freelancer versus Unemployed/homemaker	−0.038	0.380	0.919	−0.011
Student versus Unemployed/homemaker	0.037	0.337	0.913	0.016
Employed versus Unemployed/homemaker	−0.127	0.380	0.739	−0.045
**Indirect and total effects of knowledge on consumption frequency**				
**Indirect: knowledge → risk concern → consumption frequency**	**−0.147**	**0.040**	**<0.001**	**−0.140**
**Total effect of knowledge on consumption frequency**	**−0.144**	**0.060**	**0.017**	**−0.137**

*Note:* Bold values indicates significance at p value less than 0.05.

## 4 Discussion

This study aimed to assess levels of knowledge, attitudes, and practices regarding energy drink consumption among adults in Iraq. Particular attention was given to perceived health effects, sources of information, and support for public health regulations such as warning labels and age restrictions. A conceptual model was also tested in which higher knowledge was linked to a latent “risk concern” construct, which in turn was linked to past 12‐month use and consumption frequency. Overall, the findings suggest that energy drinks are widely used, particularly among younger adults. Participants reported high awareness of potential health risks and strong support for regulation, but there was a clear gap in practical knowledge about ingredients and product content.

Just over one third of participants reported energy drink use in the past year, and most users reported infrequent intake. This is broadly consistent with pooled international evidence showing that energy drink consumption is common, particularly among young people, but varies widely by setting and sampling frame [3]. The age profile of the sample (predominantly 18–25 years) likely contributes to the observed prevalence and is consistent with prior Iraqi studies reporting substantial use among young adults and university students [4, 5]. Importantly, frequency alone does not capture risk. Potential harm is shaped by patterns of use, including episodic high intake, having multiple drinks in one session, and timing of consumption, particularly late‐day use or use to stay awake for extended periods [10]. Mixing energy drinks with alcohol was uncommon in this sample, but it remains clinically relevant because experimental evidence identified greater stimulation without reversal of alcohol‐related response‐inhibition impairment [16]. In a multinational student study, risk‐taking was primarily associated with alcohol intake rather than energy‐drink mixing itself [11]. Clinical guidance nevertheless identifies co‐consumption as a pattern requiring attention [10].

Among past‐year energy drink consumers, palpitations or an irregular heartbeat and sleep disturbance were the most commonly reported symptoms after consumption. These reports were consistent with the broader literature, in which cardiovascular and sleep‐related outcomes are central concerns [7, 8, 10]. Systematic reviews indicate that energy drink intake is associated with measurable cardiovascular changes and symptoms such as palpitations, as well as sleep disruption and related neurobehavioral effects, although the size and clinical importance of these effects vary by dose, individual susceptibility, and co‐exposures [7, 8]. Lower reporting of liver or kidney “stress” is also unsurprising, as these effects are difficult to identify without medical assessment. A small study of female university students examined renal and liver biochemical markers in relation to consumption [9]. Such findings provide context for questions about perceived organ‐related harm, although biochemical markers and participants’ perceptions are distinct from diagnosed organ disease.

A central contribution of the study is the combination of high recognition of health risks and low awareness of ingredients. Participants showed strong knowledge of several widely discussed harms, including increased blood pressure, arrhythmia‐related symptoms, sleep problems, and risks linked to daily use, yet fewer than half reported knowing what energy drinks contain. This reflects a broader pattern in the literature. Consumers often understand that energy drinks can be harmful but are less clear about why specific products carry risk, how caffeine intake accumulates across servings, and how combined ingredients may increase stimulant effects [2, 10, 35]. This matters for prevention because general awareness of risk may not lead to safer behavior unless it can be applied to everyday choices. For example, it helps to know the caffeine content of a typical can and what multiple cans in a single session may mean physiologically. The findings therefore support educational approaches that go beyond generic warnings and build ingredient and dose literacy in plain language. Safety guidance also highlights that vulnerability differs by age and body size, with lower suggested thresholds for children and adolescents than for healthy adults, which supports the emphasis on youth vulnerability and the value of age‐targeted communication [36].

Participants’ attitudes strongly favored regulation, with high endorsement of warning labels, public awareness campaigns, and age restrictions. This is consistent with regional studies among adolescents and university students, which often report high perceived harm and support for public health measures even when consumption is common [26, 27, 28]. In Iraq, where energy drinks are widely marketed and readily available [4], these attitudes suggest a receptive context for policy and communication initiatives. This may be particularly true for measures that are visible at the point of purchase, such as warning labels, and for approaches that address digital exposure and marketing claims. The consistency between these findings and earlier work in neighboring settings suggests that concerns about youth, sleep, and cardiovascular effects may provide shared and credible starting points for public messaging across the region [26, 27, 28]. It also indicates that public support for regulation is unlikely to be the main barrier. Instead, the key challenge may be effective implementation and delivering messages that are specific enough to guide everyday choices.

The SEM findings describe the associations of knowledge and risk concern with consumption. In the full sample, higher knowledge was associated with higher risk concern, and higher risk concern was associated with a lower likelihood of past‐year use. At the same time, knowledge showed a small direct positive association with past‐year consumption, while the indirect pathway through risk concern was negative and statistically significant. One possible interpretation is that knowledge among consumers is related to their familiarity with energy drinks, social media content, peer discussion, or personal experience of adverse effects. The inverse association of risk concern with consumption is consistent with clinical and public health discussions of perceived risk and vulnerability in relation to avoidance and reduction, particularly when information feels relevant and credible [10]. In this context, knowledge may partly reflect involvement with energy drinks.

The drinkers‐only model adds an important practical nuance. Among past‐year consumers, risk concern was again inversely associated with consumption frequency, and the indirect association of knowledge through risk concern was negative. Notably, the total association of knowledge with frequency was also negative and statistically significant. This suggests that among people who already consume energy drinks, greater knowledge and concern may be linked to moderation rather than abstention. This distinction matters because harm reduction may be a more realistic and measurable public health goal for adult consumers than cessation, particularly when energy drinks are used to support studying, long working hours, or maintaining alertness, motivations that are commonly described in consumer and market profile research [2]. Evidence linking energy drink use to sleep outcomes in student populations also supports the value of moderation‐focused targets, such as reducing late‐day intake or limiting multi‐can sessions, since even non‐daily use can be associated with poorer sleep parameters [14].

The demographic patterns in the structural models are consistent with existing evidence and can inform targeting. In the full sample, male gender, smoking, and urban residence were associated with a higher likelihood of past‐year use, while older age was associated with a lower likelihood of use. Clinical discussions identify the co‐occurrence of energy drink use with smoking and alcohol use as relevant when considering vulnerability and potential harm [10]. These findings suggest that prevention and education efforts in Iraq may benefit from a dual approach. Broad strategies could focus on improving ingredient and dose literacy and strengthening labeling and marketing protections, alongside more targeted efforts for groups with higher likelihood of use, such as younger men and smokers, and in settings where access and marketing exposure are likely to be greater.

### 4.1 Strengths, Limitations, and Future Directions

Several strengths increase confidence in the contribution of the study. The questionnaire was developed from prior research [26, 27, 28], underwent expert content validation, followed established forward–backward translation principles [29], and was piloted before full deployment. Analytically, the study moved beyond descriptive reporting by using CFA to support the measurement structure and SEM to test construct‐level pathways, including indirect associations, which provides a more coherent account of how knowledge and concern may jointly relate to behavior.

Limitations should be considered when interpreting the findings. The cross‐sectional design precluded establishing temporal ordering or causality, and participants’ self‐reported symptoms and perceived health problems were not clinically verified. Eligibility relied on self‐reported age and residence, and respondents’ identities were not independently verified despite the account‐based submission restriction and checks for response consistency and potential duplication. Convenience sampling via social media likely over‐represented younger, urban, and more educated adults, limiting generalizability to older adults and those with limited internet access, although younger adults are the main target of this study due to their higher energy drink consumption. In addition, while the study assessed frequency and typical session volume, it did not estimate caffeine dose or identify brands, which would improve risk interpretation given the wide variation in caffeine content and serving sizes across products [2] and the centrality of dose in safety thresholds [36]. Future research would benefit from more representative sampling, more detailed exposure assessment (including caffeine dose, timing of intake, and multi‐can sessions), and longitudinal designs to examine whether ingredient literacy and risk concern are associated with subsequent changes in frequency or higher‐risk consumption patterns. Prospective evidence syntheses also suggest that adolescent energy drink consumption can precede later substance use, supporting the importance of early preventive action and of restricting youth access where feasible [37].

## 5 Conclusion

This study shows that energy drink consumption is common among adults in Iraq, particularly among younger adults, even though most users reported infrequent intake. Participants demonstrated strong awareness of several potential health risks and expressed high support for regulatory measures such as warning labels, public awareness campaigns, and age restrictions. At the same time, practical knowledge about ingredients and product content was limited, indicating an important gap in “product literacy” alongside high general risk awareness. The structural models further suggest that knowledge is linked to higher risk concern, and that higher risk concern is linked to lower likelihood of past‐year use and lower consumption frequency among consumers. Taken together, the findings support public health approaches in Iraq that combine clearer product information with targeted education and feasible regulatory action, with particular attention to younger adults and higher‐risk groups.

## Author Contributions

**Walid Al‐Qerem:** formal analysis, writing – review and editing. **Alaa Hussein Alsajri:** conceptualization, project administration, supervision. **Anan Jarab:** writing – original draft, writing – review and editing. **Ahmed Safaa:** investigation, writing – original draft. **Eman Sattar:** investigation, writing – original draft. **Ruaa Nief:** investigation, writing – original draft. **Othman Saad:** investigation, writing – original draft. **Omar Nassr:** investigation, writing – original draft. **Israa Nather:** writing – review and editing. **Judith Eberhardt:** writing – review and editing. **Lama Sawaftah:** formal analysis, writing – original draft. **Mayar Sweidan:** formal analysis, writing – original draft.

## Funding

The authors have nothing to report.

## Conflicts of Interest

The authors declare no conflicts of interest.

## Disclosure

All authors have read and approved the final version of the manuscript. Walid Al‐Qerem and Alaa Hussein Alsajri had full access to all data in this study and take complete responsibility for the integrity of the data and the accuracy of the data analysis.

## Transparency Statement

Walid Al‐Qerem and Alaa Hussein Alsajri affirm that this manuscript is an honest, accurate, and transparent account of the study being reported; that no important aspects of the study have been omitted; and that any discrepancies from the study as planned have been explained.

## Data Availability

The data that support the findings of this study are available from the corresponding author upon reasonable request.
